# Bambara groundnut soil metagenomics data

**DOI:** 10.1016/j.dib.2020.105542

**Published:** 2020-04-14

**Authors:** Caroline Fadeke Ajilogba, Olubukola Oluranti Babalola

**Affiliations:** a Food Security and Safety Niche Area, Faculty of Natural and Agricultural Science, North-West University, Mmabatho 2735, North West Province, South Africa; b Present institution: Agricultural Research Council – Soil, Climate and Water, Division of Agrometeorology, 600 Belvedere Street, Arcadia 0083, Pretoria, South Africa

**Keywords:** Rhizosphere, Microbes, Soil, Legumes, Metagenome, DNA, Sequences

## Abstract

Metagenomics analysis was carried out on extracted DNA of Rhizospheric soil samples from Bambara groundnut. This dataset presented reports on the bacterial communities at the different growth stages of Bambara groundnut and the bulk soil. Paired-end Illumina-Miseq sequencing of 16S rRNA genes was carried on the soil samples of the bacterial community with the phyla dominated by Actinobacteria (30.1%), Proteobacteria (22%), Acidobacteria (20.9%), Bacteroides (8.4%), Chloroflex (4.5%) and Firmicutes (4.4%) in all the soil samples. Samples from the bulk soil had the least average percent phyla, while samples at seed maturity stage had the highest average percent phyla. The alpha diversity at *p* = 0.05 was highest at this stage compared to the others and the control. Rubrobacter was the most predominant genera, after which is Acidobacterium and Skermanella. The biodiversity profile generated from the metagenomics analysis is useful in increasing knowledge of the drought-tolerance ability of Bambara groundnut. The data generated can be used to compare bacterial diversity at different growth stages of plants.

Specifications table

**Table:** 

Subject	Ecology, molecular biology, soil science
Specific subject area	Applied Microbiology and Biotechnology, metagenomics, microbial ecology, soil microbiology
Type of data	Figure
	Dataset of rhizospheric bacterial diversity
How data were acquired	Field data were collected by soil sampling during a 2- year growing season (2014–2016). DNA extraction from soil samples was carried out using MOBIO PowerSoil® DNA Isolation Kit (MO BIO Laboratories, Inc., Carlsbad, CA, USA), PCR was carried out in a 28 cycle PCR (5 cycles used on PCR products) using the HotStar Taq Plus Master Mix Kit (Qiagen, USA). Sequencing was performed at MR DNA (www.mrdnalab.com, Shallowater, TX, USA) on a MiSeq following the manufacturer's guidelines. Sequence data were processed using MR DNA analysis pipeline (MR DNA, Shallowater, TX, USA)
Data format	Raw
Parameters for data collection	Metagenomics DNA extraction data. Sequenced data were derived by sequencing the V3 V4 region of the 16S rRNA gene as described at MR DNA Laboratory (www.mrdnalab.com).
Description of data collection	Sequencing was done using Illumina Miseq, they were joined together and cleaned by removing barcodes and primers. Sequences less than 150 bp and sequences with ambiguous base calls were removed
Data source location	Institution: North-West University, Mafikeng Campus
	City/Town/Region: Mmabatho/Mafikeng/NorthWest
	Country: South Africa
	Latitude and longitude (and GPS coordinates) for collected samples/data: Lat. 25.82 S Long. 25.61 W
Data accessibility	Repository name: NCBI SRA
	Data identification number: Bambara groundnut rhizosphere Metagenomics data deposited in SRA as BioProject ID: PRJNA422360 and samples were given BioSample accession: SAMN12662353-SAMN12662362
	Direct URL to data: https://www.ncbi.nlm.nih.gov/biosample/8176610

## Value of the data

• As a neglected and an underutilized crop, Bambara groundnut is drought tolerant, exudates from the plants are found in the soil, making the soil a compendium of compounds and genes that need to be explored, developed and used for increase in crop production, food security and also climate change mitigation. This is because the latest generation of high throughput screening of the 16S rRNA gene shows bacterial diversity of soil samples, and high bacterial diversity ultimately boosts the production of crops. Resident plants influence and reorganize rhizospheric microbial communities through root exudates, which provides nutrients for microbial communities and control microbial diversity. The reduction of microbial diversity can affect the recycling of nutrients in the soil.
• Drought-tolerant bacteria *Rubrobacter* spp was the most abundant species in the rhizosphere of Bambara groundnut from this dataset using the high throughput screening of the 16S rRNA gene. The need to produce crops that can withstand climate change will make these data important for genetic engineering in multidisciplinary researches by the agronomist, plant breeders, soil and crop scientist, agrometeorologists, crop modelers
• These data give insight and contribution to the diversity of microbes in the soil rhizosphere of the Bambara groundnut such that some of the organisms in the profile can be used in various biocontrol and biofertilizer application.

## 1 Data

**Fig. 1:**
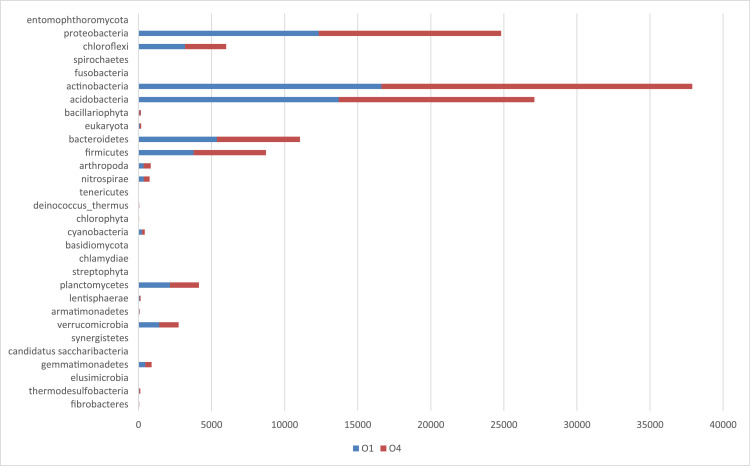
Major bacterial phyla present in the bulk soil (Soil samples O1 and O4) of Bambara groundnut as detected using the next generation sequencing (NGS).

**Fig. 2:**
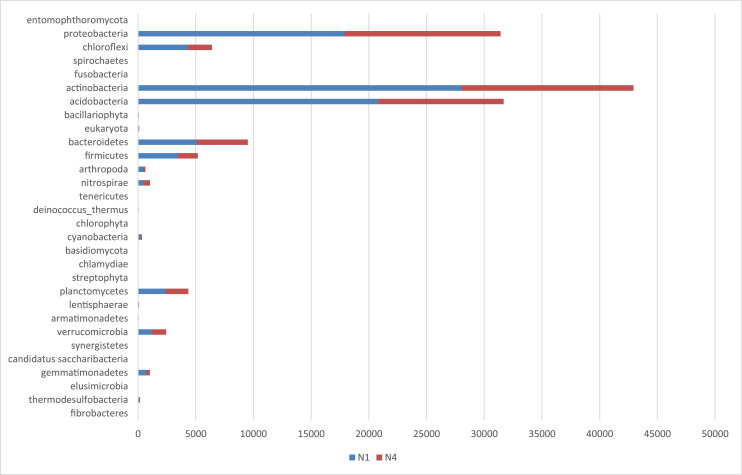
Major bacterial phyla of rhizospheric soil of 4 weeks after planting (WAP) of Bambara groundnut (Soil samples N1 and N4) as detected using the next generation sequencing (NGS).

**Fig. 3:**
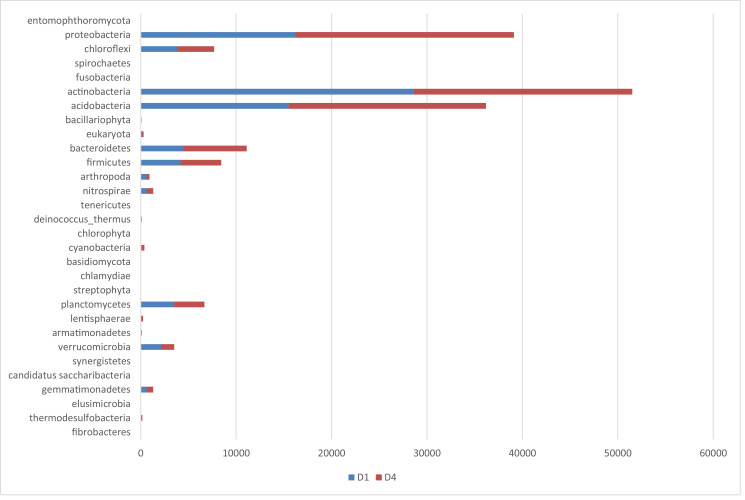
Major bacterial phyla of rhizospheric soil of 8 weeks after planting (WAP) of Bambara groundnut (Soil samples D1 and D4) as detected using the next generation sequencing (NGS).

**Fig. 4:**
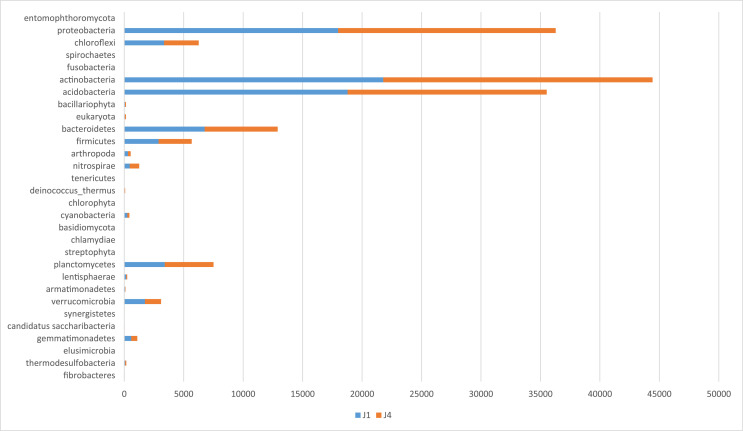
Major bacterial phyla of rhizospheric soil of 12 weeks after planting (WAP) of Bambara groundnut (Soil samples J1 and J4) as detected using the next generation sequencing (NGS).

**Fig. 5:**
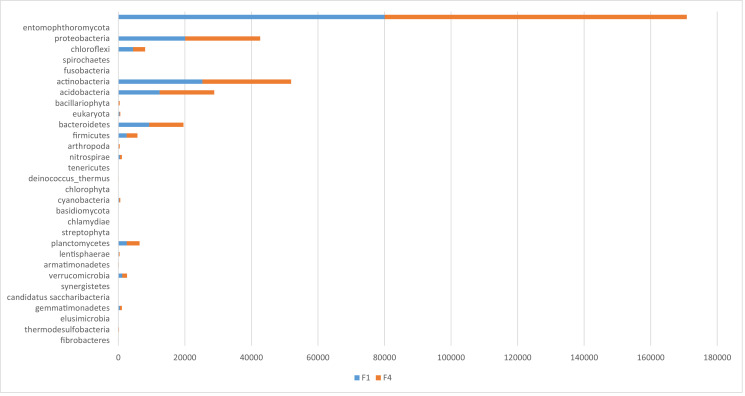
Major bacterial phyla of rhizospheric soil of 16 weeks after planting (WAP) of Bambara groundnut (Soil samples F1 and F4) as detected using the next generation sequencing (NGS).

The metagenomics dataset shows the diversity of microbes in the rhizosphere of Bambara ground at different growth stages. The raw data files were deposited in the National Center for Biotechnology Information (https://www.ncbi.nlm.nih.gov/) under accession number: SAMN12662353- SAMN12662362 and BioProject ID: PRJNA422360. O1 and O4 are the bulk soil before planting started. N1, N4; D1, D4; J1, J4; F1, F4 are data from soil samples after 4 weeks of planting (WAP), 8 WAP, 12 WAP, and 16 WAP. The composition of each sample was illustrated in Figs 1–5. The abundance of bacterial phyla in each sample was also shown in Table 1.

**Table 1:** Soil samples at different growing season showing the abundance of bacterial phyla in each sample.

Soil samples	Phyla
F1	80,055
F4	90,818
D1	80,880
D4	88,362
J1	78,881
J4	77,119
N1	85,229
N4	52,286
O1	60,373
O4	65,899

## 2 Experimental design, materials, and methods

### 2.1 Collection of soil samples from Bambara groundnut root rhizosphere

Soil samples were collected from field trials during the planting period between October 2014 and March 2016 from the North-West University agricultural farm, Mafikeng campus (Lat. 25°82′18″ Long. 25°61′44̋”) Mafikeng, South Africa [1]. Apart from the bulk soil that was collected before planting, four soil samples each were collected randomly from the uprooted Bambara groundnut root rhizosphere at four weeks interval for 16 weeks corresponding to different growth stages of the plant. Twenty-four (24) soil samples were collected in all to a depth of 15 cm per sample and were stored at 4 °C until ready for use.

### 2.2 DNA extraction from soil samples

DNA extraction from soil samples was carried out using MOBIO PowerSoil® DNA Isolation Kit (MO BIO Laboratories, Inc., Carlsbad, CA, USA) and following the manufacturer's instructions. Soil samples (0.25) g from the Bambara groundnut rhizosphere were added to the powerbead tubes and gently vortexed to mix the components of the powerbead which should have helped to lyse and disperse the soil particles. Sixty (60) µl of solution C1 was added to the powerbead tube, inverted and vortexed at maximum speed for 10 min. The powerbead tube was then centrifuged at 10,000 x g for 30 s at room temperature. The supernatant is transferred into a 2 ml collection tube provided. Two hundred and fifty (250) µl of solution C2 was added to the solution in the powerbead and vortexed for 5 s after which they were incubated at 4 °C for 5 min. The tubes were centrifuged at room temperature for 1 min at 10,000 x g. Six hundred (600) µl of supernatant was transferred to a clean 2 ml collection tube and the pellets were avoided while transferring. Two hundred (200) µl of solution C3 was added, vortexed briefly and incubated at 4 °C for 5 min. The tubes were then centrifuged at room temperature for 1 min at 10,000 x g. Up to 750 µl of supernatant were transferred into each clean 2 ml collection tube. Solution C4 was shaken to mix it before adding 1.2 ml to the supernatant and vortexed for 5 s. Approximately 675 µl of supernatant was loaded onto a spin filter and centrifuged at 10,000 x g for 1 min at room temperature. The flow-through was discarded and another additional 675 µl were added and the process was repeated thrice for each sample. Five hundred (500) µl of solution C5 were added to the spin filter and centrifuged at room temperature for 30 s at 10,000 x g. This was to help clean the DNA that was bound to the silica filter membrane and the flow-through was discarded from the 2 ml collection tube. The spin filter was then centrifuged at room temperature for 1 min at 10,000 x g and placed in a clean 2 ml collection tube. One hundred (100) µl of solution C6 were added to the center of the white filter membrane to elute the DNA. This was also centrifuged at room temperature for 30 s at 10,000 x g. Finally, the spin filters were discarded and the DNA collected in the collection tube. DNAs in the collection tube from all samples were kept frozen until further analysis to be performed at Molecular Research DNA laboratory (MR DNA, Shallowater, Texas, USA).

### 2.3 PCR amplification of bambara groundnut soil 16S rRNA gene

PCR primers 515/806 with barcoding on the forward primer were used in a 28 cycle PCR (5 cycles used on PCR products) using the HotStar Taq Plus Master Mix Kit (Qiagen, USA) to target the 16S rRNA gene for region V3 and V4. The following conditions; 94 °C for 3 min, followed by 28 cycles of 94 °C for 30 s, 53 °C for 40 s and 72 °C for 1 min, after which a final elongation step at 72 °C for 5 min was used to perform the PCR amplification.

### 2.4 Data processing sequences

In other to determine the success of amplification, PCR products were checked in 2% agarose gel to determine the relative intensity of bands. Illumina DNA library was prepared by using the pooled and purified PCR products. This was done by pooling multiple samples in equal proportions on their molecular weight and DNA concentrations. Pooled samples were purified using calibrated Ampure XP beads. Sequencing was performed at MR DNA (www.mrdnalab.com, Shallowater, TX, USA) on a MiSeq following the manufacturer's guidelines. Sequence data were processed using the MR DNA analysis pipeline (MR DNA, Shallowater, TX, USA).

### 2.5 Sequence analytical pipeline

Sequenced data were derived by sequencing the V3–V4 region of the 16S rRNA gene as described at MR DNA Laboratory (www.mrdnalab.com). Sequences were joined together and cleaned by removing barcodes and primers, sequences less than 150 bp and sequences with ambiguous base calls. Homopolymer runs exceeding 6 bp were also removed from the data set. Sequences were then denoised, operational taxonomic units (OTU's) were generated after which chimeric sequences and all abnormal sequences were removed. Filtered species-level OTUs were defined by clustering at a 3% divergence (97% similarity). Finally, these OTUs were taxonomically classified using BLASTn [2,3] against a curated database derived from RDPII and NCBI (www.ncbi.nlm.nih.gov, http://rdp.cme.msu.edu) and compiled by taxonomic level into both “counts” and “percentage” files. Sequences were considered to be at the species level if they have more than 97% identity to annotated rRNA gene sequence. They were considered to be at the genus level, family level, order level, class level, and phylum level if the sequences have identities between 95 and 97%; between 90 and 95%; between 85 and 90%; between 80 and 85%; and those between 77 and 80% respectively [4].

### 2.6 Statistical and diversity analysis

All statistical analyses were run in the statistical environment R (version 2.15.0) [5], and significant differences were defined at P values of <0.05. Alpha diversity (measures of microbiota diversity within each soil sample) was calculated using the Shannon index of diversity (H) and Simpson index. Evenness was calculated based on the Shannon index calculations, and richness was based on taxonomic identities in each sample. These were performed using the “vegan” package in R. Beta diversity (measures of microbiota differences and similarities among soil samples from growth stages) was also carried out to measure the association matrix using Bray-Curtis. The overall structure of microbial communities was ordinated by principal coordinate analysis (PCoA) based on the Bray distance metric.

## Appendix Supplementary materials

Image, application 1
